# Fine‐Tuning of Sirtuin 1 Expression Is Essential to Protect the Liver From Cholestatic Liver Disease

**DOI:** 10.1002/hep.30275

**Published:** 2019-01-29

**Authors:** Britt A. Blokker, Monica Maijo, Marta Echeandia, Mikel Galduroz, Angela M. Patterson, Anna Ten, Mark Philo, Rebecca Schungel, Virginia Gutierrez‐de Juan, Emina Halilbasic, Claudia Fuchs, Gwenaelle Le Gall, Malgorzata Milkiewicz, Piotr Milkiewicz, Jesus M. Banales, Simon M. Rushbrook, José M. Mato, Michael Trauner, Michael Müller, María Luz Martínez‐Chantar, Marta Varela‐Rey, Naiara Beraza

**Affiliations:** ^1^ Norwich Medical School University of East Anglia Norwich United Kingdom; ^2^ Gut Microbes and Health Institute Strategic Programme Quadram Institute Norwich United Kingdom; ^3^ Metabolomics Unit Quadram Institute Norwich United Kingdom; ^4^ Department of Food, Nutrition, Facilities University of Applied Sciences Münster Münster Germany; ^5^ CIC bioGUNE, Centro de Investigación Biomédica en Red de Enfermedades Hepáticas y Digestivas (CIBERehd) Derio Spain; ^6^ Hans Popper Laboratory of Molecular Hepatology, Division of Gastroenterology and Hepatology, Department of Internal Medicine III Medical University Vienna Vienna Austria; ^7^ Department of Medical Biology Pomeranian Medical University Szczecin Poland; ^8^ Liver and Internal Medicine Unit, Department of General, Transplant and Liver Surgery Medical University of Warsaw Warsaw Poland; ^9^ Department of Liver and Gastrointestinal Diseases Biodonostia Health Research Institute, Donostia University Hospital, University of the Basque Country (UPV/EHU), CIBERehd, Ikerbasque Donostia Spain; ^10^ Department of Gastroenterology Norfolk and Norwich University Hospital Norwich United Kingdom

## Abstract

Cholestasis comprises aetiologically heterogeneous conditions characterized by accumulation of bile acids in the liver that actively contribute to liver damage. Sirtuin 1 (SIRT1) regulates liver regeneration and bile acid metabolism by modulating farnesoid X receptor (FXR); we here investigate its role in cholestatic liver disease. We determined SIRT1 expression in livers from patients with cholestatic disease, in two experimental models of cholestasis, as well as in human and murine liver cells in response to bile acid loading. SIRT1‐overexpressing (SIRT^oe^) and hepatocyte‐specific SIRT1‐KO (knockout) mice (*SIRT^hep–/–^*) were subjected to bile duct ligation (BDL) and were fed with a 0.1% DDC (3,5‐diethoxycarbonyl‐1,4‐dihydrocollidine) diet to determine the biological relevance of SIRT1 during cholestasis. The effect of NorUDCA (24‐norursodeoxycholic acid) was tested in BDL/SIRT^oe^ mice. We found that SIRT1 was highly expressed in livers from cholestatic patients, mice after BDL, and Mdr2 knockout mice (*Mdr2^–/–^*) animals. The detrimental effects of SIRT1 during cholestasis were validated *in vivo* and *in vitro*. SIRT^oe^ mice showed exacerbated parenchymal injury whereas *SIRT^hep–/–^* mice evidenced a moderate improvement after BDL and 0.1% DDC feeding. Likewise, hepatocytes isolated from SIRT^oe^ mice showed increased apoptosis in response to bile acids, whereas a significant reduction was observed in *SIRT^hep–/–^* hepatocytes. Importantly, the decrease, but not complete inhibition, of SIRT1 exerted by norUDCA treatment correlated with pronounced improvement in liver parenchyma in BDL/SIRT^oe^ mice. Interestingly, both SIRT1 overexpression and hepatocyte‐specific SIRT1 depletion correlated with inhibition of FXR, whereas modulation of SIRT1 by NorUDCA associated with restored FXR signaling. *Conclusion:* SIRT1 expression is increased during human and murine cholestasis. Fine‐tuning expression of SIRT1 is essential to protect the liver from cholestatic liver damage.

**Abbreviations**
ALT: alanine aminotransferase
AMPK: 5' adenosine monophosphate‐activated protein kinase
ANOVA: analysis of variance
AP: alkaline phosphatase
AST: aspartate aminotransferase
BDL: bile duct ligation
Bsep: bile salt export pump
CA: cholic acid
CCL2: C‐C motif chemokine ligand 2
CCR: CC‐type chemokine receptor
CDCA: chenodeoxycholic acid
CK19: cytokeratin 19
CLD: cholestatic liver disease
*Cyp7A1*: cholesterol 7 hydroxylase
DCA: deoxycholic acid
DDC: 3,5‐diethoxycarbonyl‐1,4‐dihydrocollidine
FXR: farnesoid X receptor
GCA: glycocholic acid
IFNγ: interferon‐gamma
IHC: immunohistochemistry
IL: interleukin
KO: knockout
Mrp4: multidrug resistance‐associated protein 4
NMCs: normal mouse cholangiocytes
norUDCA: 24‐norursodeoxycholic acid
NorUDCA/SIRT1^oe^: overexpressing mice that have been treated with NorUDCA
NorUDCA: Nor‐ursodeoxycholic acid
NOS2: nitric oxide synthase 2
Ntcp: sodium taurocholate cotransporting polypeptide
Oatp: organic anion transporting polypeptide
PBC: primary biliary cholangitis
PSC: primary sclerosing cholangitis
SHP: small heterodimer partner
SIRT1: Sirtuin 1
αSMA: alpha‐smooth muscle actin
TNFα: tumor necrosis factor alpha
TUNEL: terminal deoxynucleotidyl transferase dUTP nick end labeling
UDCA: ursodeoxycholic acid
WT: wild type

The term cholestatic liver disease (CLD) includes a broad spectrum of aetiologically heterogeneous hepatobiliary disorders, mainly comprising primary biliary cholangitis (PBC) and primary sclerosing cholangitis (PSC), in adults. These conditions are characterized by accumulation of bile acids in the liver, leading to hepatocellular necrosis and apoptosis, progressive fibrosis, and end‐stage liver disease.^1^, ^2^, ^3^ Current therapeutic approaches for treating cholestasis mainly rely on the use of ursodeoxycholic acid (UDCA); however, this treatment has no proven efficacy for PSC and a proportion of patients with PBC.^2^, ^3^ The therapeutic options for such unresponsive patients are currently limited, though there have been recent promising advances, including the use of 24‐norursodeoxycholic acid (NorUDCA),^4^ which has been shown to improve liver function in PSC patients in a recent clinical trial.^5^ Also, novel treatments using fibrates^6^ and farnesoid X receptor (FXR) agonists, such as obeticholic acid,^7^, ^8^, ^9^ have shown efficacy for PBC patients unresponsive to UDCA. Still, a better understanding of the molecular mechanism underpinning the pathogenesis of cholestasis will enable the development of efficient therapies for cholestatic patients.

FXR is an orphan nuclear receptor that plays a key role in the regulation of bile acid metabolism and in the pathogenesis of cholestasis.^6^, ^10^, ^11^, ^12^, ^13^ Regulation of FXR involves a dynamic acetylation/deacetylation process mediated by p300 and Sirtuin 1 (SIRT1), respectively.^14^ SIRT1 deacetylates FXR, increasing its DNA binding and dependent gene transcription. Interestingly, SIRT1/FXR interaction must be finely tuned, given that prolonged SIRT1‐mediated FXR deacetylation leads to ubiquitination and proteasome degradation.^14^

SIRT1 is an evolutionarily conserved nicotinamide adenine dinucleotide^+^–dependent histone III deacetylase that is activated in response to energy deprivation, controlling key metabolic functions, including bile acid metabolism.^15^, ^16^ Initial work delineating the implication of SIRT1 in prolonging the life span in lower organisms^17^ and in promoting healthy aging in mammals^18^ led to SIRT1 being hyped as a “magic bullet” to preserve lifelong health. Nevertheless, the role of SIRT1 has been revealed to be highly complex in a wide range of biological functions, including tumorigenesis. We and others have described SIRT1 as being highly expressed in human liver tumors,^16^, ^19^, ^20^ pointing to the potential contribution of SIRT1 to liver disease. Supporting this, we demonstrated that SIRT1 overexpression leads to impaired liver regeneration after partial hepatectomy, which associated with disturbances in bile acid homeostasis, including reduced FXR signaling, increased synthesis and accumulation of toxic bile acids in the liver.^16^ Overall, these results led us to hypothesise that SIRT1 may play a role during CLD.

In accord, in this study, we suggest that SIRT1 is up‐regulated in the liver during human cholestasis in PSC and PBC patients and in two murine models of cholestasis; after bile duct ligation (BDL) and in Mdr2 knockout mice (*Mdr2^–/–^*) mice. We further demonstrate that SIRT1 contributes to liver parenchymal damage in the context of obstructive cholestasis, given that overexpression of SIRT1 aggravates liver injury, whereas hepatocyte‐specific SIRT1 depletion exerts a moderate cell protection after BDL and feeding with a diet containing 0.1% of DDC (3,5‐diethoxycarbonyl‐1,4‐dihydrocollidine). Importantly, the improvement in liver function observed in hepatocyte‐specific SIRT1 KO (knockout) mice is only transient, likely involving mechanisms including the attenuation of FXR signaling. Ultimately, we describe that the beneficial effect of NorUDCA treatment in reducing liver injury in cholestatic SIRT1‐overexpressing mice associates with the modulation, though not complete depletion, of SIRT1 expression.

Overall, our results support the importance of maintaining SIRT1 fine‐tuned expression to preserve liver function in the context of cholestatic disease.

## Materials and Methods

### HUMAN PBC AND PSC SAMPLES

SIRT1 gene expression was determined by qPCR analysis in mRNA isolated from cirrhotic livers of patients with PBC (n = 10) and PSC (n = 10) who underwent liver transplantation. Control liver tissues (n = 5) were acquired from large‐margin liver resections from patients undergoing of colorectal metastases with no microscopic changes of liver disease identified by a pathologist, all collected in the Department of General, Transplant and Liver Surgery, Medical University of Warsaw (Warsaw, Poland), as described elsewhere.^21^ Supporting Table S1 includes detailed clinical and biochemical data of these patients.

Protein expression of SIRT1 was assessed by immunohistochemistry (IHC) in paraffin‐embedded sections from livers obtained by percutaneous biopsy from n = 9 PBC patients, n = 5 PSC patients, and in liver samples obtained from n = 4 healthy individuals at the Norwich Norfolk University Hospital (Norwich, UK). The diagnosis was established by pathological analysis of liver biopsies together with presence of antimitochondrial antibodies in the case of PBC. Clinical and biochemical data of these patients are included in Supporting Table S2. Use of human tissue samples was approved by the Faculty of Medicine and Health Sciences Research Ethics committee (University of East Anglia, Norwich, UK). Collection and handling of human samples used in this study conformed to the Declaration of Helsinki and the Human Tissue Act (UK) and Good Clinical Practice Guidelines (UK).

### EXPERIMENTAL PROCEDURES IN ANIMALS

All experimental procedures were conducted in male mice from 8‐12 weeks of age and were performed at the CICbioGUNE animal facility and at the Disease Modelling Unit (University of East Anglia, UK) and were previously approved by the Department of Environment, Planning, Agriculture and Fisheries (Basque Country Government, Spain) and by the Animal Welfare and Ethical Review Body (AWERB, University of East Anglia, Norwich, UK) respectively. All experiments were performed following the guidelines of the National Academy of Sciences (National Institutes of Health publication 86‐23, revised 1985) and were conducted within the provisions of the Animals (Scientific Procedures) Act 1986 (ASPA) and the LASA Guiding Principles for Preparing for and Undertaking Aseptic Surgery (2010) under UK Home Office approval.

More information is available in the Supporting Materials and Methods.

### STATISTICAL ANALYSIS

Data are expressed as mean ± standard error of the mean. Statistical significance was determined by two‐way analysis of variance (ANOVA) followed by a Student’s *t* test or by a Student’s *t* test only as appropriate using Graph Pad Prism software.

## Results

### SIRT1 IS UP‐REGULATED DURING HUMAN AND MURINE CHOLESTASIS

**Figure 1:**
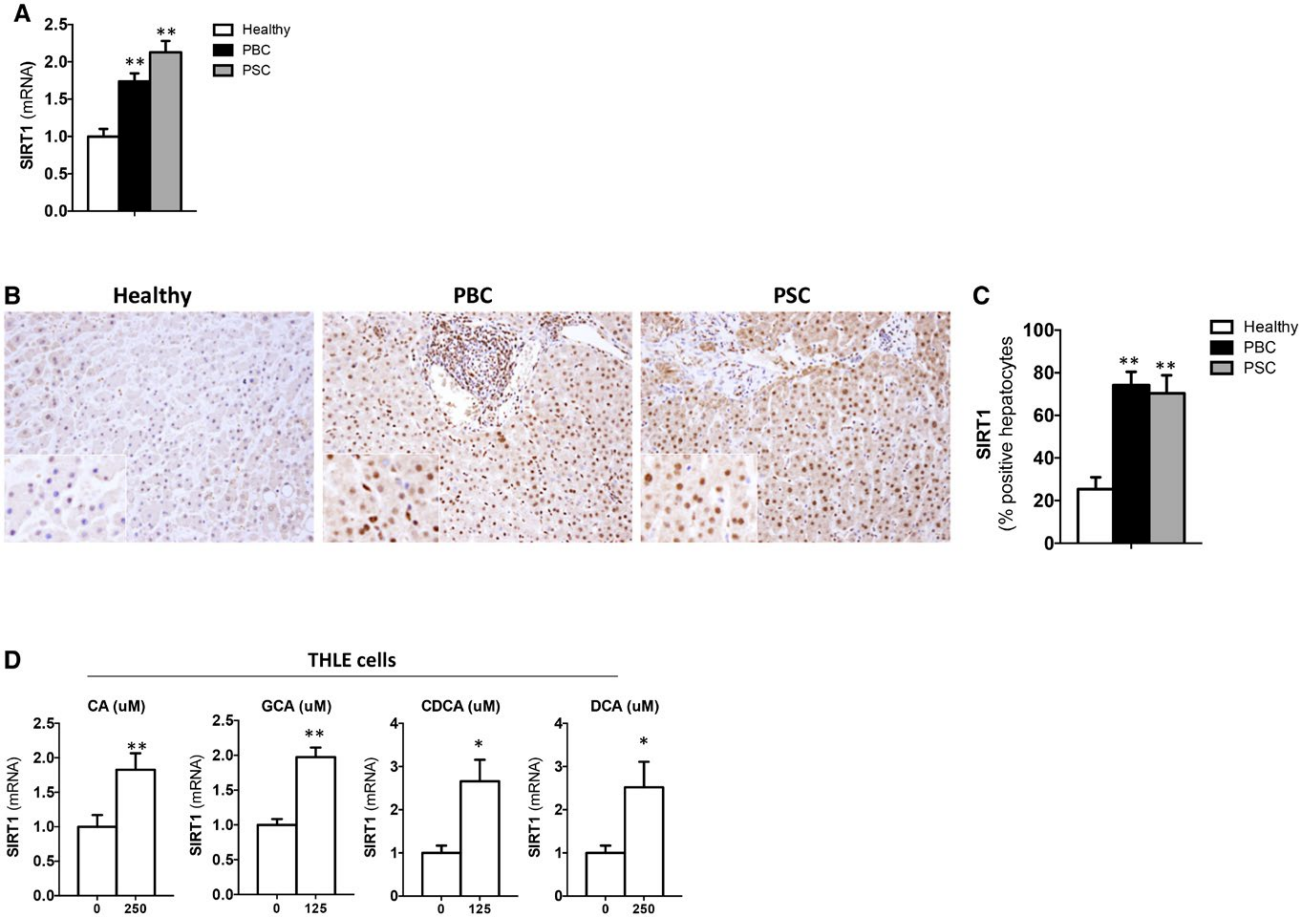
SIRT1 is highly expressed in livers from cholestatic PBC and PSC patients and is induced in response to bile acids *in vitro*. (A) qPCR analysis of SIRT1 expression in liver samples from healthy individuals (n = 5), PBC (n = 10), and PSC (n = 10) and (B) IHC using an anti‐SIRT1 Ab in liver sections from cholestatic patients compared to healthy donors showing increased gene and protein expression and protein nuclear localization of SIRT1 in hepatocytes and cholangiocytes during cholestasis (original magnification, ×10) with (C) quantification of positively stained nuclei. Healthy individuals, n = 4; PBC, n = 9; PSC, n = 5. (D) qPCR analysis of SIRT1 expression in THLE2 cells cultured for 3 hours with CA, GCA, CDCA, and DCA and at the doses indicated. Values are mean ± SEM; *in vitro* experiments were performed three times in triplicate; *P < 0.05; **P < 0.01. Abbreviation: Ab, antibody

Expression of SIRT1 during PBC and PSC, the main human CLD etiologies, has not been characterized to date. SIRT1 was highly expressed in cholestatic livers from PBC and PSC patients at the gene transcript level (Fig. 1A). IHC analysis evidenced increased positive SIRT1 immunostaining mainly localized in the nuclei of hepatocytes and bile duct cells in PBC and PSC patients (Fig. 1B,C). In contrast, lower and more‐diffuse SIRT1 staining was detected in livers from healthy individuals (Fig. 1B,C). These results suggest that increased SIRT1 nuclear expression relates to the cholestasis itself and not to the specific etiology of the disease.

To determine whether bile acids have a direct effect on triggering SIRT1 up‐regulation during cholestasis, we exposed THLE‐2 cells (liver epithelial cells of human origin) to different bile acids, including primary and secondary species, and found a significant increase in SIRT1 expression (Fig. 1D).

**Figure 2:**
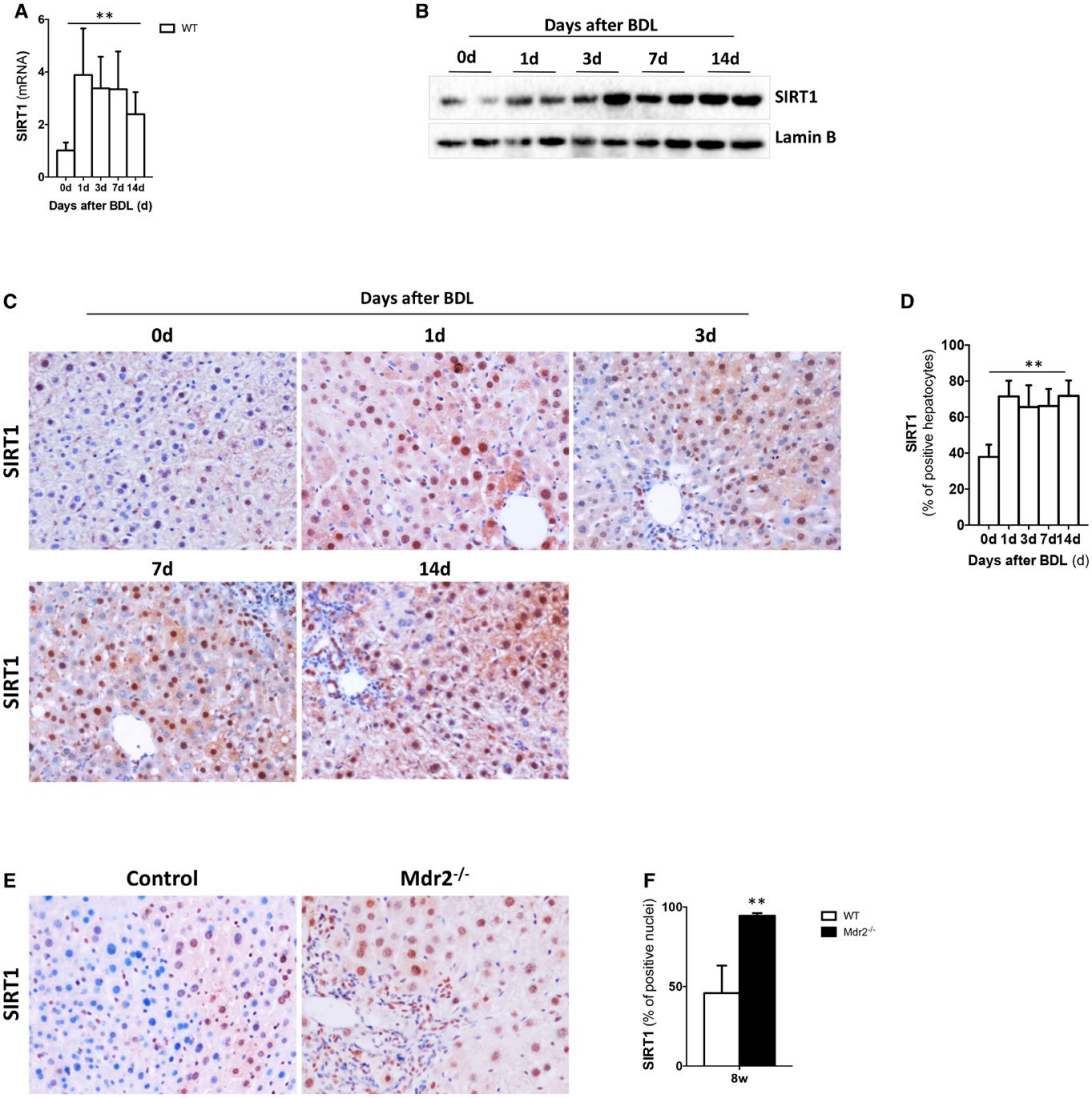
SIRT1 expression is up‐regulated during surgically and genetically induced murine cholestasis. (A) qPCR analysis of SIRT1 expression in livers from WT mice at different time points after BDL showing up‐regulation during cholestasis. (B) Western blotting analysis on liver nuclear extracts from WT mice and (C) IHC on liver sections and (D) further quantification of SIRT1‐positive nuclei after BDL, indicating increased SIRT1 expression and nuclear localization during cholestasis. (E) IHC in liver sections of WT and *Mdr2^–/–^* mice and (F) quantification of SIRT1‐positive nuclei. Values are mean ± SEM; n ≥ 5 animals/time point; **P < 0.01.

Further studies in murine models of cholestasis confirmed that SIRT1 is up‐regulated at different time points after BDL at gene (Fig. 2A) and protein level (Fig. 2B‐D and Supporting Fig. S1A) in wild‐type (WT) mice (Fig. 2C,D). No changes in SIRT1 expression were observed in livers from sham‐operated mice (Supporting Fig. S1B,C).

In accord with our results in mice after BDL, analysis of liver tissue samples from *Mdr2^–/–^* mice, a well‐established mouse model resembling PSC,^22^ showed an increased number of hepatocytes expressing SIRT1, as evidenced by IHC and further quantification of positive hepatocytes (Fig. 2F), and higher protein expression in nuclear liver extracts, as shown by immunoblotting analysis (Supporting Fig. S1D,E).

*In vitro* studies in primary hepatocytes from WT mice supported our observations in human liver cells (Fig. 1D), showing SIRT1 up‐regulation in response to chenodeoxycholic acid (CDCA), deoxycholic acid (DCA), glycocholic acid (GCA), and cholic acid (CA) at a dose of 125 µM (Supporting Fig. S2A). Increased SIRT1 expression in hepatocytes associated with augmented apoptosis after bile acid load (Supporting Fig. S2B) was not altered in the presence of caspase‐3 inhibitor (Supporting Fig. S2C), supporting that SIRT1 up‐regulation is not resulting from increased apoptosis. Further studies using the bile acid species with a higher impact on cell death showed that CDCA and DCA triggered 5' adenosine monophosphate‐activated protein kinase (AMPK) phosphorylation (Supporting Fig. S2D). Inhibition of AMPK activity partially reduced SIRT1 expression (Supporting Fig. S2E) and decreased apoptosis (Supporting Fig. S2F). SIRT1 and AMPK are key metabolic regulators activated in response to changes in nutrient or energy availability.^23^, ^24^ Importantly, serum supplementation to the culture media reduced AMPK phosphorylation (Supporting Fig. S2G), SIRT1 expression (although still present; Supporting Fig. S2H), and apoptosis (Supporting Fig. S2I) in response to bile acids.

Overall, our results indicate that SIRT1 expression increases during cholestasis, likely driven by accumulation of bile acids, contributing to hepatocyte cell death.

### SIRT1 OVEREXPRESSION AGGRAVATES LIVER INJURY, INFLAMMATION, AND FIBROGENESIS AFTER BDL

To gain further insight into the biological relevance of increased SIRT1 expression during CLD, we performed BDL in mice that overexpress SIRT1 (Supporting Fig. S3A,B; hereafter, SIRT^oe^ mice).

**Figure 3:**
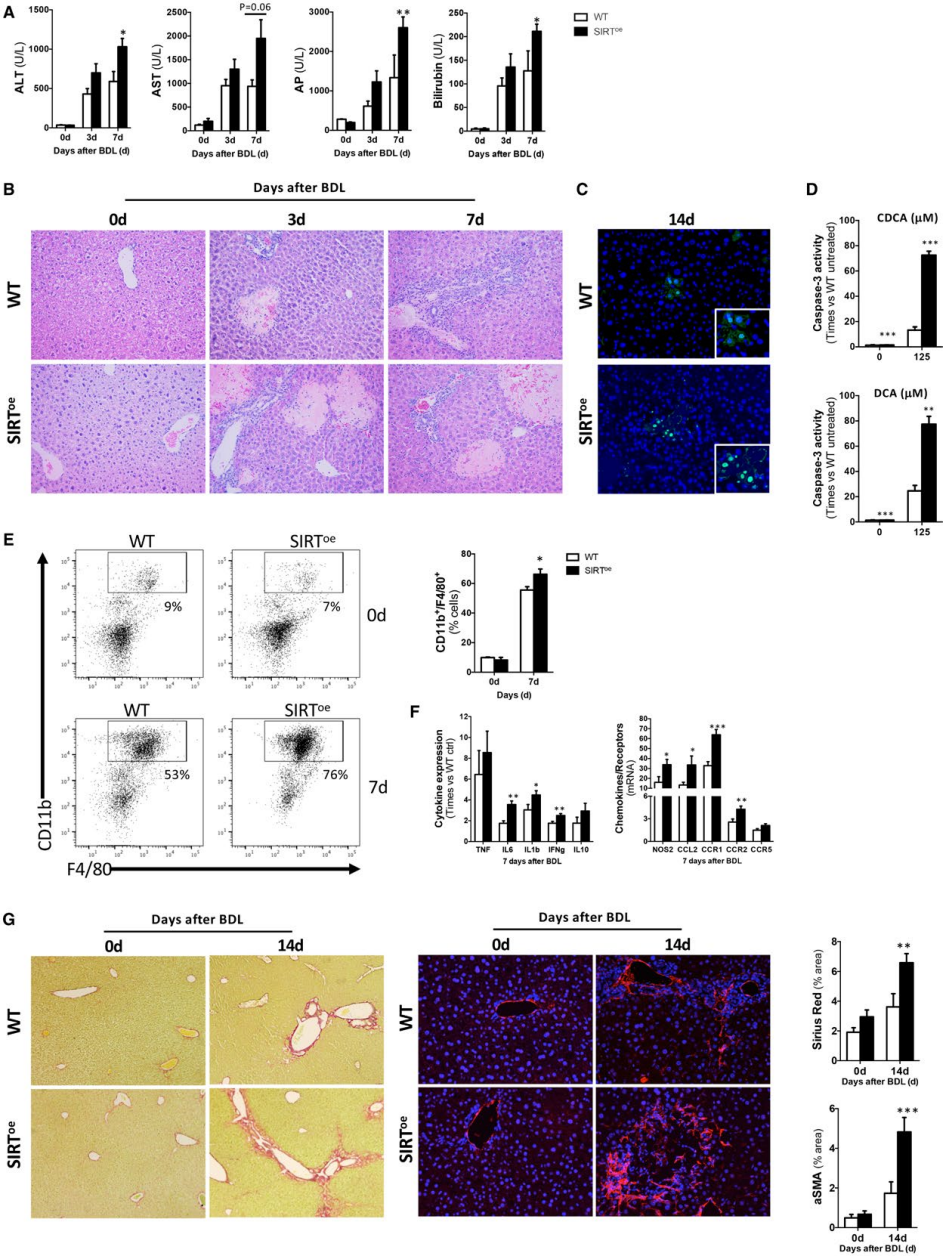
Overexpression of SIRT1 leads to increased parenchymal injury and fibrogenesis in mice after BDL. (A) Profiles of blood liver injury markers detected in WT and SIRT^oe^ animals and (B) H&E staining of liver sections from WT and SIRT^oe^ animals after BDL showing profuse liver damage in SIRT^oe^ mice. (C) TUNEL assay on liver sections showing increased presence of apoptotic hepatocytes in SIRT^oe^ mice compared to WT after BDL. (D) Caspase‐3 activity was determined in primary hepatocytes isolated from WT and SIRT^oe^ mice and cultured in the presence of CDCA and DCA. (E) FACS analysis on liver isolated immune cells and (F) qPCR analyses of inflammation markers showed increased presence of macrophages and increased proinflammatory response in SIRT^oe^ mice. (G) Liver fibrogenesis was characterized by Sirius Red staining on liver sections (left panels) and αSMA IHC (right panels) from mice after BDL, followed by morphometric quantification using Frida software expressed in % of positive staining per power field (ppf). Images are representative of n ≥ 5 animals/time point; values are mean ± SEM; *P < 0.05; **P < 0.01; ***P < 0 .001 (WT vs. SIRT^oe^). Abbreviations: ALT, alanine aminotransferase; AP, alkaline phosphatase; AST, aspartate aminotransferase.

The increase in serum markers of liver function and the profuse presence of necrotic areas observed in SIRT^oe^ mice evidenced the detrimental impact of SIRT1 overexpression during cholestasis (Fig. 3A,B). Analysis of caspase‐3 activity (Supporting Fig. S3C) and terminal deoxynucleotidyl transferase dUTP nick end labeling (TUNEL) assay (Fig. 3C and Supporting Fig. S3D) supported that higher apoptotic cell death occurs after BDL in SIRT^oe^ mice compared to WT animals.

*In vitro* analyses in isolated hepatocytes from SIRT^oe^ mice confirmed that overexpression of SIRT1 further sensitizes liver cells to bile‐acid–induced apoptotic cell death (Fig. 3D and Supporting Fig. S3E).

Characterization of inflammatory response by fluorescent‐activated cell sorting (FACS) analysis of liver‐isolated immune cells showed that SIRT^oe^ mice had higher presence of macrophages at 7 days after BDL compared to WT mice (Fig. 3E). Analysis of cytokine (interleukin [IL]1β, IL6, and interferon‐gamma [IFNγ]), activation factors (nitric oxide synthase 2; NOS2), chemokine (C‐C motif chemokine ligand 2; CCL2), and chemokine receptor (CC‐type chemokine receptor [CCR]1, CCR2, and CCR5) expression confirmed the increased proinflammatory milieu in SIRT^oe^ mice compared to WT after BDL. Tumor necrosis factor alpha (TNFα) expression was comparable in both genotypes (Fig. 3F). TNFα, IL6, and CCL2 enzyme‐linked immunosorbent assay (ELISA) confirmed the gene expression results obtained (Supporting Fig. S3F).

Finally, fibrogenesis was assessed in SIRT^oe^ and WT mice after BDL. Sirius Red staining (Fig. 3G and Supporting Fig. S3G, left panels), alpha‐smooth muscle actin (αSMA) determination by IHC on liver sections (Fig. 3G and Supporting Fig. S3G, right panels), followed by quantification (Fig. 3G) and qPCR analysis of collagen 1A1, αSMA, and transforming growth factor beta gene expression (Supporting Fig. S3H) supported increased fibrogenesis in SIRT^oe^ animals compared to WT mice after BDL.

Analysis of WT and SIRT^oe^ mice at 3 and 7 days after sham surgery showed no significant differences compared to control animals in the parameters described above, including serum liver damage markers (Supporting Fig. S4A), liver parenchyma status (Supporting Fig. S4B), hepatocyte apoptosis (Supporting Fig. S4C), inflammation (Supporting Fig. S4D,E), and fibrosis (Supporting Fig. S4F,G). Macrophage counts and caspase‐3 were slightly increased in sham WT and SIRT^oe^ mice, respectively, compared to control mice, though these parameters were still significantly different in BDL mice, supporting the specificity of the biological response observed after BDL.

Overall, our results suggest that SIRT1 overexpression aggravates liver injury, hepatocellular death, inflammation, and consequent fibrogenesis in the context of cholestasis.

### SIRT1 OVEREXPRESSION ALTERS FXR‐MEDIATED REGULATION OF BILE ACID SYNTHESIS

During cholestasis, FXR mediates compensatory responses aiming at inhibiting endogenous bile acid production and regulating their transport, in a coordinated manner with other nuclear receptors.^12^

**Figure 4:**
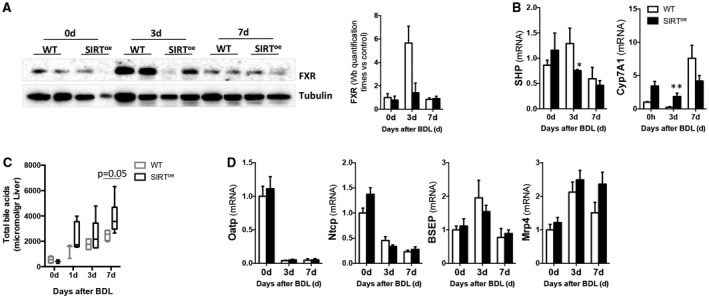
Overexpression of SIRT1 correlates with lower presence and activity of FXR and higher accumulation of bile acids in liver. (A) Western blotting of whole‐protein extracts using FXR Ab and further quantification using Image Lab software (Bio‐Rad, Hercules, CA) showing reduced protein presence in SIRT^oe^ mice. (B) Gene expression analysis of SHP and *Cyp7a1* by qPCR in livers 3 and 7 days after BDL. (C) Quantification of bile acid pool size in livers from WT and SIRT mice after BDL by HPLC. (D) qPCR analysis of bile acid transporters after BDL. Values are mean ± SEM; n ≥ 5 animals/time point; *P < 0.05; **P < 0.01 (WT vs. SIRT^oe^). Abbreviations: Ab, antibody; HPLC, high‐performance liquid chromatography.

In line with the described cross‐talk regulation,^12^, ^13^, ^14^ we found that SIRT1 overexpression associates with decreased FXR protein expression during cholestasis. Whereas WT mice showed a transient increase in FXR at 3 days after BDL that ameliorated after 7 days, SIRT^oe^ mice had persistently lower FXR levels (Fig. 4A). Lower small heterodimer partner (SHP) and higher cholesterol 7 hydroxylase (*Cyp7A1*) expression found in SIRT^oe^ mice compared to WTs at 3 days after BDL confirmed the impaired FXR signaling in the context of SIRT1 overexpression at this time point (Fig. 4B).

Further analysis of liver bile acid content showed an enlarged pool size in SIRT^oe^ mice after BDL compared to WT animals (Fig. 4C) whereas no significant differences were detected in fecal excretion (Supporting Fig. S5A). Analysis of bile acid transporters showed no significant differences between WT and SIRT^oe^ mice in organic anion transporting polypeptide (Oatp), sodium taurocholate cotransporting polypeptide (Ntcp), bile salt export pump (Bsep), or multidrug resistance‐associated protein 4 (Mrp4) expression (Fig. 4D), supporting that increased bile acid accumulation in SIRT^oe^ mice resulted from higher synthesis.

Sham surgery had no impact on modulating FXR signaling or bile acid transporter expression compared to untreated control mice, with the exception of Ntcp, of which expression was reduced in sham mice compared to control animals, but was still significantly different from WT/BDL and SIRT^oe^/BDL mice (Supporting Fig. S5B‐D).

Overall, our results demonstrate that SIRT1 overexpression contributes to the accumulation of bile acids in the liver during cholestasis upon attenuation of FXR‐mediated inhibition of bile acid synthesis.

### SIRT1 OVEREXPRESSION ATTENUATES CHOLANGIOCYTE PROLIFERATION

Liver cholestasis is characterized by chronic bile duct injury with proliferation of cholangiocytes (ductular reaction) at the early stages and later ductopenia.^1^, ^3^

**Figure 5:**
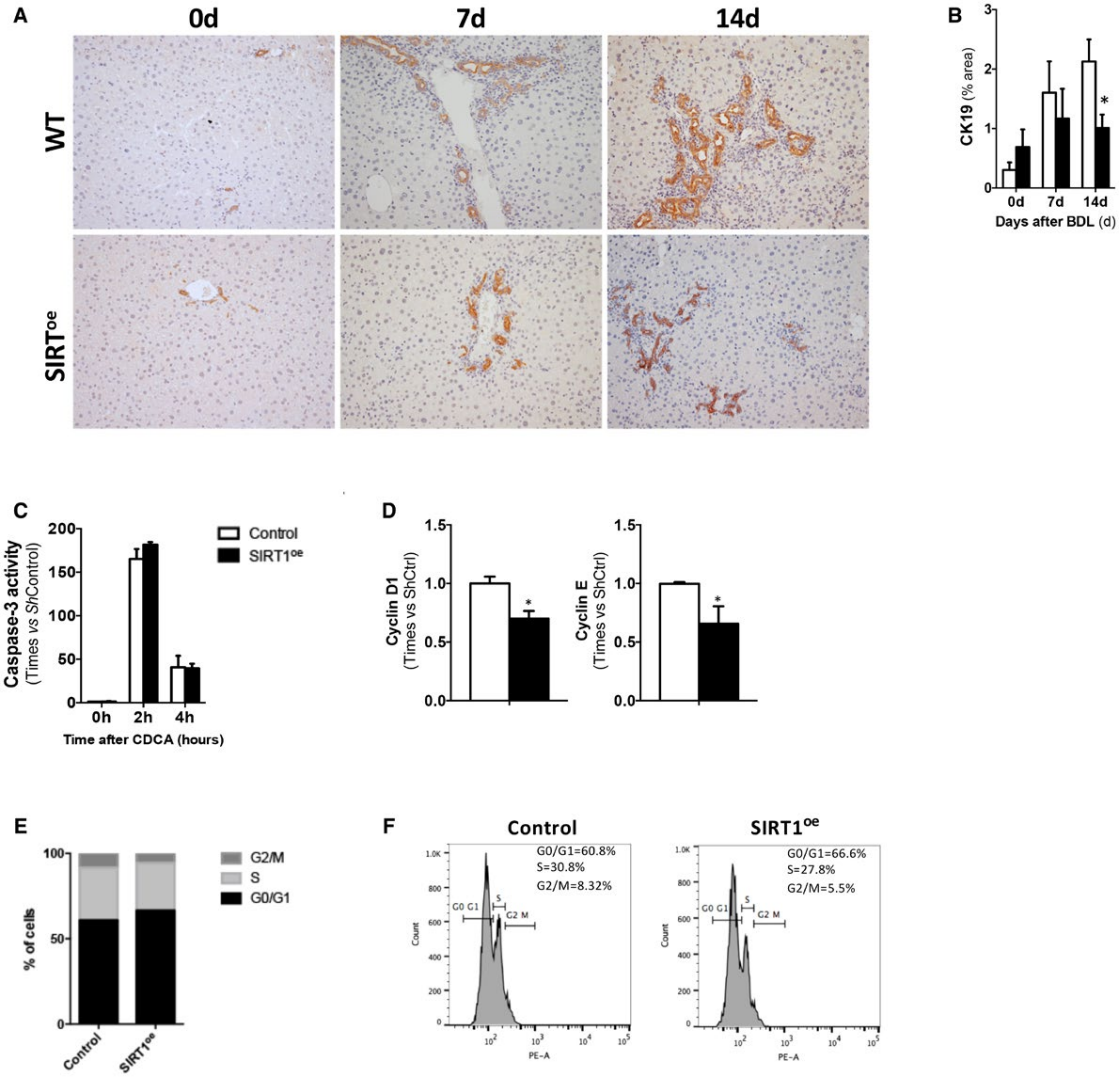
SIRT1 overexpression correlates with attenuation of cholangiocyte proliferation. (A) IHC using an anti‐CK19 Ab in paraffin‐embedded liver sections at different time points after BDL and (B) further quantification using Frida software, expressed in % of positive staining ppf (original magnification, ×10) showing milder ductular reaction in SIRT mice compared to WTs. (C) Determination of caspase‐3 activity in response to CDCA on NMCs transfected with control c‐Flag pcDNA3 (Ctrl) or pCruzHA SIRT1 plasmid DNA to induce overexpression of SIRT1 (SIRT^oe^). (D) qPCR analysis of cell‐cycle–related gene expression in Ctrl and SIRT^oe^ transfected NMC showing lower proliferation in the presence of growth factors (EGF) 36 hours after transfection. (E) FACS analysis of PI‐stained NMCs confirming lower numbers of cells in S phase after SIRT1 overexpression compared to control transfected cells. (F) Representative histograms after FACS analysis of NMC transfected cells in culture. Values are mean ± SD; n = 5 animals/time point; *in vitro* experiments were performed three times in triplicate; *P < 0.05; **P < 0.01 (WT vs. SIRT); (Ctrl vs. SIRT1^oe^). Abbreviations: Ab, antibody; EGF, epidermal growth factor; PI, propidium iodide; ppf, per power field; ShCtrl, short hairpin control.

Interestingly, cytokeratin 19 (CK19) immunostaining on liver sections showed a moderate increase of the ductular reaction in WT compared to SIRT^oe^ mice (Fig. 5A,B).

Aiming to determine how SIRT1 may influence cholangiocyte function, we performed *in vitro* analysis of normal mouse cholangiocytes (NMCs), where we induced overexpression of SIRT1 by transfecting with a plasmid DNA (SIRT1^oe^). An empty vector was transfected as control (Control). We found that SIRT1^oe^/NMC showed similar apoptotic response to bile acid stimulation when compared to control/NMC (Fig. 5C). Further analysis of cell‐cycle regulation revealed that SIRT1^oe^ NMC had lower cyclin D1 and E expression when cultured in the presence of growth factors, suggesting that SIRT1 overexpression may attenuate cell proliferation (Fig 5D). Finally, FACS analysis, showing a higher percentage of SIRT1^oe^/NMC arrested in the G_1_ phase compared to control cells (Fig. 5E,F), confirmed that SIRT1 overexpression attenuates cholangiocyte proliferation.

### SIRT1 OVEREXPRESSION CONTRIBUTES TO INCREASED LIVER INJURY AND FIBROGENESIS AFTER 0.1% DDC–INDUCED CHOLESTASIS

The detrimental impact of SIRT1 overexpression during cholestasis was further confirmed in an additional experimental model where 0.1% DDC–fed SIRT^oe^ mice showed increased alkaline phosphatase (AP) serum levels (Supporting Fig. S6A) and wider areas of liver necrosis after 1 week of treatment (Supporting Fig. S6B). Though not prominent, 0.1% DDC/SIRT^oe^ showed increased cell death (Supporting Fig. S6C,D) compared to 0.1% DDC/WT animals. As observed after BDL, SIRT^oe^ mice showed milder ductular reaction, as evidenced by CK19 immunostaining (Supporting Fig. S6E) and higher fibrosis (Supporting Fig. S6F) than WT animals after DDC. Western blotting analysis showed strong reduction of FXR in both genotypes after DDC diet, although expression was found to be lower in SIRT^oe^ mice compared to WT animals (Supporting Fig. S6G).

Overall, our results in this alternative model of cholestasis support the detrimental impact that SIRT1 overexpression has on liver damage during cholestasis.

### HEPATOCYTE‐SPECIFIC SIRT1 DEPLETION LEADS TO A MODERATE, BUT TRANSIENT, ATTENUATION OF CHOLESTATIC LIVER INJURY AFTER BDL

Our results indicate that SIRT1 overexpression contributes to aggravation of liver damage during cholestasis, pointing to modulation of SIRT1 as a therapeutic approach. Next, we aimed to investigate how hepatocyte‐specific SIRT1 depletion may impact on liver injury during BDL‐induced cholestasis.

**Figure 6:**
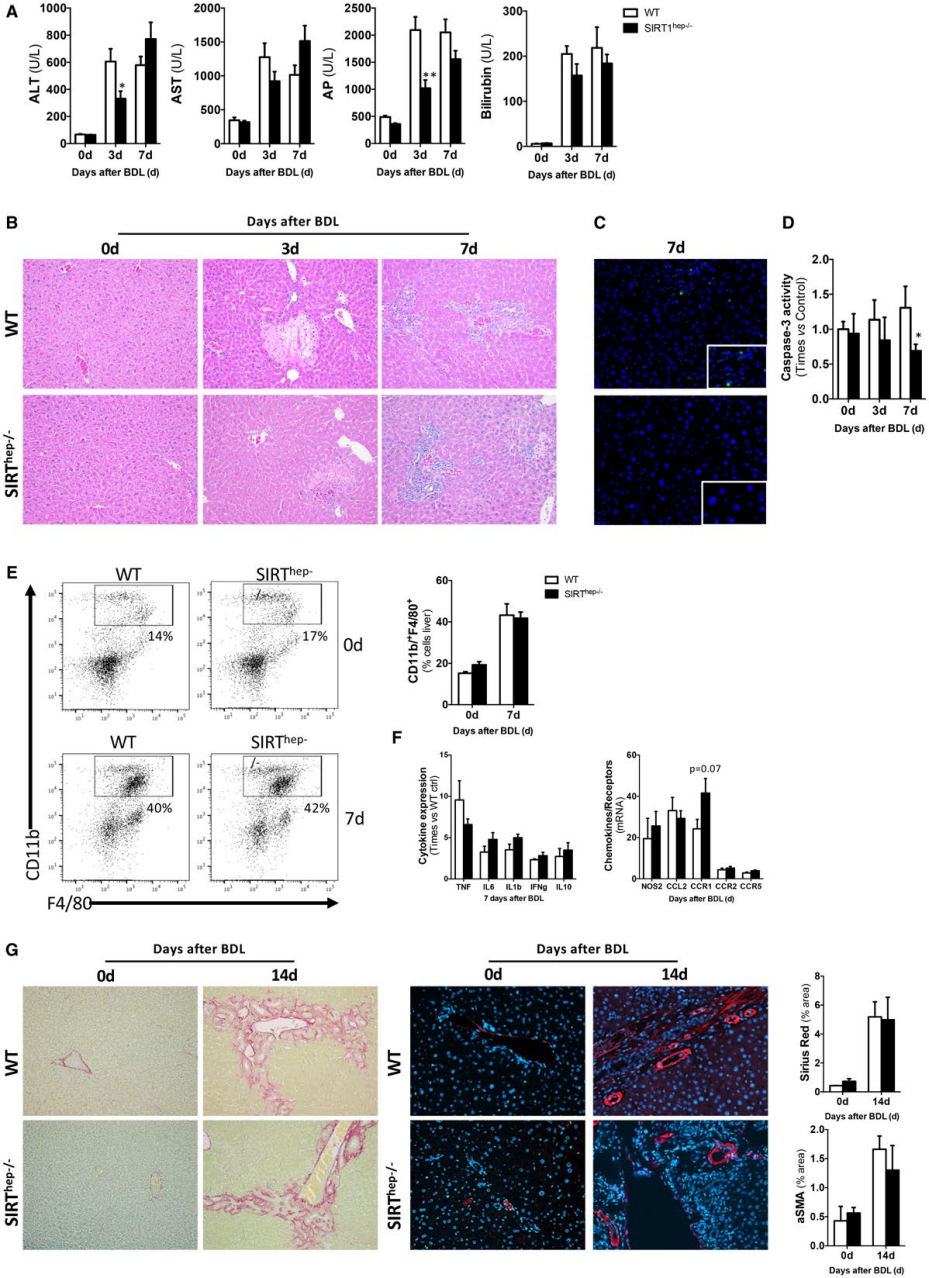
Hepatocyte‐specific SIRT1 depletion has a transient effect on protecting the liver from cholestatic injury by modulating apoptotic cell death whereas it has no impact on liver inflammation, ductular reaction, and fibrosis. (A) Levels of serum liver damage markers of WT and *SIRT^hep–/–^* animals and (B) H&E staining of liver sections from WT and *SIRT^hep–/–^* animals after BDL showing transient improvement of the damaging liver phenotype in *SIRT^hep–/–^* mice compared to WT. (C) TUNEL assay on liver sections showing decreased presence of apoptotic hepatocytes in *SIRT^hep–/–^* mice compared to WT after BDL. (D) Quantification of caspase‐3 activity and in CDCA‐ and DCA‐treated primary hepatocytes isolated from WT and *SIRT^hep–/–^* mice showing a decrease apoptosis in *SIRT^hep–/–^* mice, indicating an increase in necrosis. (E) FACS analysis on liver isolated immune cells and (F) ELISA on liver extracts showed comparable presence of macrophages and cytokine production in *SIRT^hep–/–^* and WT mice. (G) Liver fibrogenesis was characterized by Sirius Red staining on liver sections (left panels) and aSMA IHC (right panels) from mice after BDL followed by quantification using Frida software expressed in % of positive staining per power field (ppf). All images at original magnification 10×. Values are mean ± SEM; n ≥ 5 animals/time point; *in vitro* experiments were performed three times in triplicate; *P < 0.05; **P < 0.01 (WT vs. *SIRT^hep–/–^*).

We found that hepatocyte‐specific SIRT1 KO mice (*SIRT^hep–/–^*) with only residual SIRT1 expression in the liver (Supporting Fig. S7A,B) showed a moderate improvement on liver damage markers and liver parenchymal status (Fig. 6A,B), whereas liver injury seemed to reach comparable levels as WT mice at later time points (7 days) after BDL (Fig. 6A,B). Quantification of apoptotic response by TUNEL assay (Fig. 6C and Supporting Fig. S7C) and caspase‐3 activity (Supporting Fig. S7D) showed a reduction of apoptotic cell death in *SIRT^hep–/–^* mice compared to WT mice after BDL.

*In vitro* analysis confirmed that hepatocytes isolated from *SIRT^hep–/–^* mice had a significantly lower apoptotic response to DCA and CDCA (Fig. 6D), whereas overall cell survival was lower in KO cells when compared to WT cells (Supporting Fig. S7E), indicating an increase in necrosis. Interestingly, hepatocytes isolated from *SIRT^hep–/–^* mice showed reduced phosphorylated AMPK levels (Supporting Fig. S7F) after bile acid stimulation, supporting the cross‐talk between SIRT1 and AMPK in response to bile acids. In line with the reduction in apoptosis upon SIRT1 depletion, inhibition of AMPK blunted bile‐acid–induced apoptosis (Supporting Fig. S7G).

Liver inflammation was comparable in *SIRT^hep–/–^* mice and WT animals after BDL given that there were no significant differences in liver macrophages (Fig. 6E) or in expression TNF, IFNγ, IL10, NOS2, CCL2, CCR2, and CCR5 (Fig. 6F and Supporting Fig. S8A). The mild increase in IL6, IL1β, and CCR1 in *SIRT^hep–/–^* mice was not statistically significant. We found no significant differences in ductular reaction (Supporting Fig. S8B,C) or degree of fibrosis (Fig. 6G and Supporting Fig. S8D,E) in *SIRT^hep–/–^* mice compared to WT animals after BDL.

**Figure 7:**
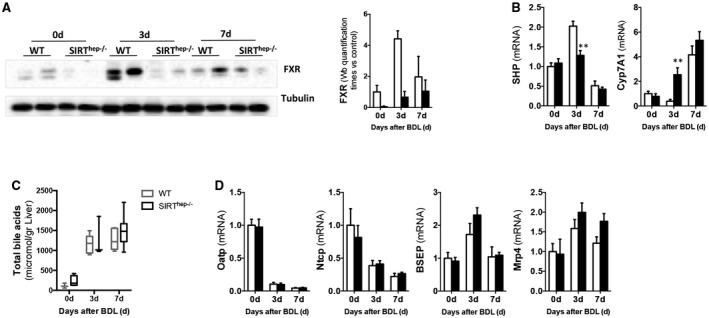
Hepatocyte‐specific SIRT1 depletion associates with reduced FXR expression and signaling after BDL and comparable bile acid transporters expression than WT littermates. (A) Western blotting of whole‐protein extracts and further quantification using ImageLab software (Bio‐Rad, Hercules, CA) showing reduced FXR in *SIRT^hep–/–^* mice after BDL. (B) Gene expression analysis of SHP and *Cyp7a1* and (C) bile acid transporters by qPCR in livers 3 and 7 days after BDL. (D) Quantification of bile acid pool size in livers from WT and *SIRT^hep–/–^* mice after BDL by MS‐HPLC. Values are mean ± SEM; n ≥ 5 animals/time point; **P < 0.01 WT versus *SIRT^hep–/–^*). Abbreviations: HPLC, high‐performance liquid chromatography; MS, mass spectrometry.

Interestingly, *SIRT^hep–/–^* mice had lower FXR protein expression 3 and 7 days after BDL compared to WT littermates (Fig. 7A). Reduced FXR correlated with lower SHP and higher *Cyp7A1* expression in *SIRT^hep–/–^* mice at 3 days after BDL that were further regulated 7 days after surgery similarly in both WT and KO mice (Fig. 7B). Accordingly, with the increased SIRT1, we found reduced FXR acetylation at 3 and 7 days after BDL in WT mice (Supporting Fig. S9A). In accord with our observations, FXR acetylation and total protein expression were further reduced in *SIRT^hep–/–^* mice (Supporting Fig. S9A). Consequently, an increased accumulation of bile acids in liver of *SIRT^hep–/–^* mice was observed after BDL (Fig. 7C), though this did not reach statistical significance. Expression of bile acid transporters was comparable between *SIRT^hep–/–^* and WT mice after BDL (Fig. 7D), supporting that increased BA synthesis is a consequence of attenuated FXR signaling in the absence of hepatocytic SIRT1 and not to changes in transport. Though not statistically significant, *Mrp4* expression was slightly higher in *SIRT^hep–/–^* mice compared to WT animals, which could reflect a slight increase in alternative transport of bile acids, overall impacting on total bile acid pool size.

Sham surgery had no impact on modulating liver injury (Supporting Fig. S10A‐C), inflammation (Supporting Fig. S10D,E), ductular reaction (Supporting Fig. S10F), and fibrosis (Supporting Fig. S10G,H) in *SIRT^hep–/–^* mice.

Overall, our results suggest that SIRT1‐hepatocyte depletion exerts a degree of protection against bile‐acid–induced apoptosis, but not necrosis, explaining the transient benefits observed during cholestasis *in vivo* in *SIRT^hep–/–^* mice.

### HEPATOCYTE‐SPECIFIC SIRT1 DEPLETION LEADS TO A MODERATE, BUT TRANSIENT, ATTENUATION OF CHOLESTATIC LIVER INJURY AFTER 0.1% DDC DIET FEEDING

Further analyses in 0.1% DDC–treated *SIRT^hep–/–^* mice confirmed our results obtained in the BDL experimental model, given that, despite the reduction in levels of liver damage serum markers in *SIRT^hep–/–^* mice compared to WTs (Supporting Fig. S11A), no significant differences in parenchyma structure were detected (Supporting Fig. S11B). Similarly to what was found after BDL, though not prominent, 0.1% DDC/*SIRT^hep–/–^* mice had lower apoptosis (Supporting Fig. S11C,D) compared to 0.1% DDC/WTs. Finally, ductular reaction and fibrosis were comparable in *SIRT^hep–/–^* and WT mice 1 week after 0.1% DDC feeding (Supporting Fig. S11E,F). Western blotting analysis showed a decrease in FXR expression in both genotypes after DDC diet that was more pronounced in *SIRT^hep–/–^* mice (Supporting Fig. S11G).

### THE BENEFICIAL EFFECTS OF NorUDCA ON ATTENUATING CHOLESTATIC LIVER INJURY ASSOCIATE WITH THE REDUCTION, BUT NOT INHIBITION, OF SIRT1 EXPRESSION

NorUDCA has proven efficacy in treating murine cholestasis in *Mdr2^–/–^* mice^25^, ^26^ and improving cholestasis in PSC patients.^5^ We previously described that NorUDCA reduced SIRT1 expression in noncholestatic SIRT^oe^ mice, which associated with an improved response to injury and restored regenerative capacity of the liver.^16^ These observations evidenced an alternative mechanism of action of this drug that may be relevant to cholestasis and lead us to investigate the impact of NorUDCA on SIRT1 expression during cholestasis.

**Figure 8:**
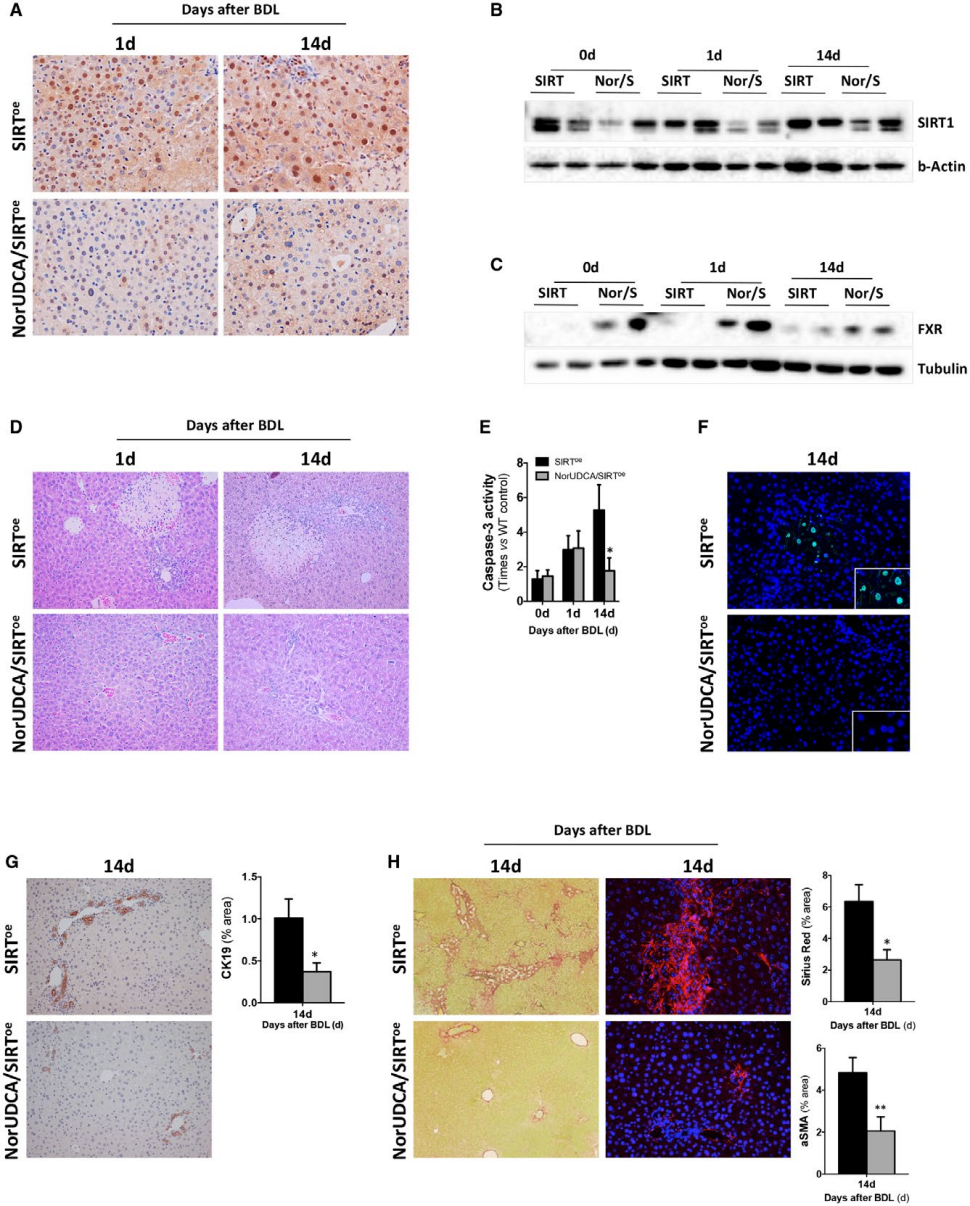
NorUDCA lowers SIRT1 expression leading to restored FXR signaling, improved liver parenchyma status, and reduced apoptosis, ductular reaction, and fibrogenesis after BDL. (A) IHC in liver sections using a SIRT1 Ab and (B) western blotting analysis showing reduction of SIRT1, but (C) sustained FXR protein expression in NorUDCA/SIRT^oe^ mice after BDL. (D) H&E staining of liver sections, (E) quantification of caspase‐3 activity on liver protein lysates, and (F) TUNEL assay on liver sections from SIRT^oe^ and NorUDCA/SIRT^oe^ mice after BDL confirmed attenuation of parenchymal injury in the latter. (G) CK19 IHC as well as (H) Sirius Red staining and aSMA IHC on liver sections, all followed by morphometric analyses, confirmed the beneficial impact of NorUDCA in BDL/SIRT^oe^ mice. All images at original magnification 10× or 20× (SIRT1). Values are mean ± SEM; n ≥ 5 animals/time point; *P < 0.05; **P < 0.01 (SIRT vs. NorUDCA/SIRT). Abbreviations: Ab, antibody; NorUDCA, nor‐ursodeoxycholic acid; NorUDCA/SIRT1^oe^ overexpressing mice that have been treated with NorUDCA.

Our results show that NorUDCA significantly reduced SIRT1 expression in SIRT^oe^ mice during cholestasis after BDL, both at the gene transcript (Supporting Fig. S12A) and protein level (Fig. 8A,B and Supporting Fig. S12B). Lower SIRT1 expression correlated with higher FXR expression in NorUDCA/BDL/SIRT^oe^ mice compared to BDL/SIRT^oe^ (Fig. 8C and Supporting Fig. S12C). Reduced bile acid pool size was detected in livers from NorUDCA/SIRT^oe^ mice after BDL compared to BDL/SIRT^oe^ animals (Supporting Fig. S8D). These changes correlated with an obvious improvement in liver parenchyma status in NorUDCA/SIRT^oe^ mice after BDL as evidenced by hematoxylin and eosin (H&E) staining (Fig. 8D), determination of serum liver injury markers (Supporting Fig. S12E), and reduced apoptotic cell death (Fig. 8E,F and Supporting. Fig. S12F). Finally, NorUDCA/SIRT^oe^ mice showed a significant attenuation of ductular reaction (Fig. 8G) and fibrogenesis (Fig. 8H and Supporting Fig. S12G) after BDL compared to SIRT^oe^ animals.

Notably, analysis of livers from *Mdr2^–/–^* mice confirmed that the described attenuation of the cholestatic phenotype exerted by NorUDCA treatment^4^ associates with the reduction of SIRT1 protein expression and nuclear localisation (Supporting Fig. S12H,I).

Taken together, our results support the importance of preserving the fine‐tuning of SIRT1 expression to protect the liver from cholestasis‐induced parenchymal injury.

## Discussion

In this study, we suggest that SIRT1 is up‐regulated in the liver during human and murine cholestasis, and that it actively contributes to liver damage in this disease context.

Our results, showing that SIRT1 expression is increased in livers from both PBC and PSC cholestatic patients regardless of the disease etiology, suggest that up‐regulation of SIRT1 may be related to accumulation of bile acids in the liver occurring during obstructive cholestasis. Previous studies evidenced that UDCA and tauroursodeoxycholic acid induce SIRT1 expression,^27^, ^28^ whereas low doses (10‐50 μM) of unconjugated species had no effect in modulating SIRT1.^28^ Here, we show that primary, conjugated, and secondary bile acids (at a dose of 125 μM) significantly induce expression of SIRT1 in human liver THLE cells and in mouse primary hepatocytes, whereas no effect was observed at lower concentrations, supporting that the dosage of bile acids is crucial to regulate SIRT1. Different doses of bile acids have a differential impact on hepatocyte physiology; whereas low concentrations of bile acids (10‐50 μM) act as signaling molecules, higher doses (from 50 to 200 μM) have a proapoptotic action.^29^, ^30^, ^31^, ^32^ In accord, our results show that up‐regulation of SIRT1 expression in response to bile acids (125 μM) correlates with apoptotic cell death in primary hepatocytes isolated from WT mice. Importantly, we found that inhibition of apoptosis had no impact on the up‐regulation of SIRT1, supporting that SIRT1 is upstream of the apoptotic response. Our additional studies confirmed the proapoptotic implication of SIRT1 up‐regulation in hepatocytes, given that apoptosis was further increased in SIRT1‐overexpressing hepatocytes in response to bile acids, whereas it was significantly reduced in SIRT1‐depleted hepatocytes compared to WT cells.

Further mechanistic *in vitro* studies pointed to the cross‐talk regulation of SIRT1 and AMPK, which is essential to mediate bile‐acid–induced cell death. Our results are in line with those in previous work showing that AMPK activation by metformin aggravated liver injury during xenobiotic‐induced cholestasis, through mechanisms involving impaired FXR signaling,^33^ and support the relevance of the SIRT1/AMPK axis in mediating bile‐acid–induced cell death.

SIRT1 and AMPK are well‐known metabolic regulators activated in response to metabolic challenges, including the decrease in cell energy levels (e.g., during starvation/fasting).^23^, ^24^ During cholestasis, disruption of the flux of bile acids to the intestine contributes to deficient lipid absorption that overall impacts on the metabolic/energy status of the liver. Importantly, work from Moustafa et al.^34^ showed that restoration of lipid metabolism in *Mdr2^–/–^* mice after NorUDCA feeding or high‐fat diet feeding protected the liver from cholestatic liver injury, pointing to the beneficial impact of increasing energy load during cholestasis. We propose that during cholestasis, the metabolic challenge involving lower nutrient/energy availability, in addition to the increase bile acid load, contribute to up‐regulation of SIRT1 and subsequent liver damage. Supporting this, our *in vitro* studies show that serum‐supplementation to culture media associated with reduced SIRT1 and AMPK activation and lower apoptosis in response to bile acids in comparison to starved hepatocytes. Although further work investigating *in vivo* activation of AMPK during cholestasis is required, our *in vitro* studies point to a role for AMPK in regulating SIRT1 and detrimental activity during cholestasis. Collectively, our results and those previously published^33^, ^34^ highlight the metabolic characteristic of cholestatic disease.

To gain further insight into the biological relevance of SIRT1 regulation during cholestasis, we performed BDL and fed SIRT1‐overexpressing mice with a 0.1% DDC diet that showed exacerbated parenchymal liver injury when compared to WT animals. Additional *in vivo* studies showed that the reduction, but not complete inhibition, of SIRT1 expression in liver had a therapeutic potential to improve liver parenchyma status during cholestasis. Thus, attenuation of liver injury in BDL/SIRT^oe^ mice after NorUDCA treatment correlated with a reduction of SIRT1 expression, whereas hepatocyte‐targeted SIRT1 depletion in *SIRT^hep–/–^* mice lead to a transient improvement in liver function that was offset at later stages after BDL. Regulation of FXR by SIRT1 may represent a key mechanism mediating these outcomes.

FXR is the main regulator of bile acid homeostasis. During cholestasis, FXR signaling mediates an adaptive response aiming to reduce bile acid pool size by inhibiting bile acid synthesis and modulating their transport.^35^ FXR currently represents a promising target for therapeutic approaches to treat human cholestatic disease.^7^, ^8^, ^9^ Regulation of FXR involves a dynamic deacetylation process coordinated by SIRT1^14^ and is needed for FXR‐DNA binding and target gene transcription, whereas the same process regulates FXR proteasomal degradation.^14^ In accord, we previously described that FXR was reduced in SIRT1‐overexpressing mice.^16^ Here, we provide further evidence of the relevance of SIRT1/FXR signaling during cholestasis. Thus, whereas SIRT1 overexpression reduced FXR signaling, attenuation of SIRT1 after NorUDCA treatment efficiently restored FXR expression after BDL. Interestingly, we also observed reduced FXR signaling in *SIRT1^hep–/–^* mice, as described in previous studies^36^ that associated with a transient reduction of liver injury after BDL, suggesting that depletion of SIRT1/FXR in hepatocytes may protect the liver at early stages of obstructive cholestasis. This is supported by previous studies,^11^, ^35^, ^37^ including work from Wagner et al., showing that whole‐body FXR‐deficient mice had lower intrabiliary pressure after BDL overall relating to less bile infarcts and attenuated liver damage after BDL.^11^ Similarly, we found reduced ductular reaction at early stages of cholestasis in both SIRT^oe^ and *SIRT1^hep–/–^* mice, though differences were not statistically significant in the latter and became comparable to WT mice at later stages after BDL.

As cholestatic disease progresses, cholangiocytes lose their proliferative capacity in advanced disease, contributing to bile duct loss (ductopenia).^3^ Our results point to the contribution of SIRT1 to this process and support the previously described ability of mild SIRT1 overexpression to inhibit the proliferative effect of growth factors like progranulin *in vitro*.^38^

Ultimately, the apparent differences in severity of the damaging phenotype observed in SIRT1‐overexpressing mice when compared to *SIRT1^hep–/–^* mice, despite the similarly attenuation of FXR signaling, support that apoptotic cell death associated with increased SIRT1 expression play a key role in contributing to liver injury during cholestasis.

Several studies using NorUDCA treatment in murine models of cholestasis,^4^, ^25^, ^39^, ^40^ and a recently conducted phase II human clinical trial,^5^ support the benefits of this drug as a treatment option for cholestatic patients.^5^, ^41^ In our present work, we show that NorUDCA modulates SIRT1 expression in two alternative models of cholestasis: in SIRT^oe^ mice after BDL and in *Mdr2^–/–^* mice. Importantly, our results show that only the modulation of SIRT1 exerted by NorUDCA, but not the complete depletion (as in our hepatocyte‐KO mice), preserved FXR signaling and overall liver function after BDL, emphasizing the relevance of maintaining fine‐tuned SIRT1 expression to protect the liver during cholestasis. In our studies, we cannot discern whether SIRT1 regulation is a mere consequence of the reduced bile acid pool size in the liver or is a direct effect of NorUDCA on SIRT1. As discussed, other factors, like restoration of liver energy metabolism upon NorUDCA treatment,^34^ may also impact on SIRT1 regulation.

Our observations are relevant to recent studies that propose the use of SIRT1 activators to counteract murine cholestasis after CA feeding in mice.^28^ It is worth noting that after bile acid feeding, SIRT1 was differently regulated than during human and murine obstructive cholestasis, where SIRT1 expression is significantly elevated. Although it is out of the scope of our current study to resolve differential SIRT1 expression during CA feeding and after BDL, previous studies have revealed marked differences between these two experimental models.^11^, ^42^ For example, CA feeding regulates intestinal pathways that feedback to control bile acid metabolism in the liver in a different way to that during obstructive cholestasis, which involves the absence of bile acids in the intestine. Hence, CA feeding commonly results in inhibition of *Cyp7A1*,^43^ likely mediated by intestinal‐derived feedback mechanisms involving activation of ileal FXR,^44^ whereas obstructive cholestasis after BDL results in initial reduction (Figs. 4 and 5), but later recovery of *Cyp7A1* expression and bile acid synthesis.^44^ Furthermore, treatment with SIRT1 activators in CA‐fed mice had no impact on SIRT1 gene expression and protein expression was only modestly induced after treatment, rendering a SIRT1 expression comparable to that found at basal homeostatic conditions.^28^ These observations ultimately support our conclusions underscoring the importance of maintaining a fine‐tuned SIRT1 expression in the liver to counteract cholestasis.

In summary, our work raises awareness that expression levels of SIRT1 should be considered when designing therapeutic strategies to treat cholestasis, which should aim to the attenuation, though not complete inhibition, of SIRT1. Overall, our results underline the critical relevance of maintaining the fine‐tuning of SIRT1 expression to preserve liver health.
