# Supplementary material for: Fine‐Tuning of Sirtuin 1 Expression Is Essential to Protect the Liver From Cholestatic Liver Disease
Source: Hepatology. 2019 Jan 29;69(2):699–716. doi: 10.1002/hep.30275 (PMC6492079; doi:10.1002/hep.30275)
Supplement: Supplementary file 1 [file HEP-69-699-s001.docx]

**SUPPLEMENTAL MATERIAL AND METHODS**

**Experimental procedures in animals**

SIRT1 overexpressing animals (SIRT^oe^) were generated on a *C57/B6J* background as described in Herranz *et al*[1]. Hepatocyte-specific SIRT1 KO mice were generated by crossing mice containing the Albumin Cre promoter with mice containing floxed sites flanking exon 4 of SIRT1 gene (B6;129-Sirt1tm1Ygu/J; Jackson Laboratories). Mice carrying loxP-flanked SIRT1 alleles were used as controls when compared to AlbCre-containing SIRT1 floxed mice. 8 week-old Mdr2^-/-^ mice (FVB/N background) were obtained from Jackson Laboratory (Bar Habor, ME) and were bred in parallel with WT mice with the same genetic background.

Cholestasis was induced by ligating the common bile duct (bile duct ligation; BDL) as previously described[2] and by feeding a subset of mice were fed with a diet containing 3,5-diethoxycarbonyl-1,4-dihydrocollidine (DDC) for 1 week. In the specific experiments, SIRT mice were fed with a diet containing 0.5% w/w 24-norursodeoxycholic acid (NorUDCA)[3-5] for 3 weeks before BDL and thereafter for the duration of the experiment.

**Materials**

Cholic, chenodeoxycholic, deoxycholic and gycocholic acid (CA, CDCA, DCA and GCA) were obtained from Sigma-Aldrich.

**Characterization of apoptotic cell death**

Apoptotic cell death was determined by quantifying Caspase-3 activity in snap frozen livers using a fluorescence-labeled substrate following the manufacturer’s instructions (Enzo).

**Mouse hepatocyte isolation**

Primary hepatocytes were isolated from WT, SIRT^oe^ and SIRT^hep-/-^ mice by perfusion of the liver and further digestion with collagenase I (Worthington). Cells were washed, pelleted and plated on rat collagen type I (BD Biosciences) pre-coated plates with Minimum Essential Medium.

***in vitro* experiments**

Human THLE-2 cells (purchased from ATCC) or freshly isolated primary mouse hepatocytes from WT, SIRT^oe^ and SIRT^hep-/-^ mice were cultured in the presence of bile acids at the doses indicated for 3 hours. mRNA was extracted and SIRT1 gene expression was analysed by qPCR.

**Bile acid determination**

Bile acids were extracted from liver samples and analyzed using high performance liquid chromatography-tandem mass spectrometry at the Quadram Institute Bioscience (QIB) Metabolomics Unit. In brief, cleaned-up extracts were analysed using HPLC-mass spectrometry operated in multiple reaction monitoring (MRM) mode. Each sample (5 µl) was analysed using an Agilent 1260 binary HPLC coupled to an AB Sciex 4000 QTrap triple quadrupole mass spectrometer. HPLC was achieved using a binary gradient of solvent A (Water + 5mM Ammonium Ac + 0.012% Formic acid) and solvent B (Methanol + 5mM Ammonium Ac + 0.012% Formic acid) at a constant flow rate of 600 µl/min. Separation was made using a Supelco Ascentis Express C18 150 x 4.6, 2.7µm column maintained at 40°C. Injection was made at 50% B and held for 2 min, ramped to 95%B at 20 min and held until 24 minutes. The column equilibrated to initial conditions for 5 minutes.

The mass spectrometer was operated in electrospray negative mode with capillary voltage of -4500V at 550°C. Instrument specific gas flow rates were 25ml/min curtain gas, GS1: 40 ml/min and GS2: 50 ml/min. Mass fragmentation was monitored and quantification was applied using Analyst 1.6.2 software to integrate detected peak areas relative to the deuterated internal standards.

**Histology, immunohistochemistry and immunofluorescence**

Liver tissues were embedded in paraffin and further sectioned, dewaxed and hydrated. Slides were stained with H&E or pathological analysis. Immunohistochemistry (IHC) was performed using anti-SIRT1[4, 6] (Santa Cruz) or CK19 (Developmental Studies Hybridoma Bank, University of Iowa) diluted in antibody diluent (Dako), after which EnVision^+^polymer HRP labelled anti-rabbit (Dako) or anti-Rat HRP labelled secondary antibody were applied respectively. IHC was developed using the DAB^+^ chromogen system (Dako) and nuclei were counterstained with hematoxylin.

Apoptotic cells were labeled using TUNEL assay (Roche) on liver sections following manufacturer’s instructions.

For SIRT1 and TUNEL, quantification of positive nuclei *vs* total nuclei per power field (ppf) was represented as a percentage. CK19 staining was quantified using Frida Software.

Fibrosis was determined with Sirius Red staining and immunofluorescence (IF) using α-smooth muscle actin (αSMA) antibody (Sigma), labeled with Cy3 (red). In this case, nuclei were counterstained with DAPI (blue). Quantification of collagen presence and αSMA were done using Frida software and are represented as the % of the stained area relative to the total area. In all cases, 5-10 fields per sample were pictured and analyzed.

**RNA isolation and Quantitative Real-Time PCR**

RNA was isolated from liver samples or cultured cells with QiAzol Reagent (Qiagen) followed by first strand synthesis with random primers and reverse transcription using M-MLV Reverse Transcriptase (Invitrogen). Quantitative real-time PCR (qPCR) was performed using SYBR Green reagent (Life technologies) in a ViiA7 Real-time PCR detection system (Applied Biosystems). Gene expression was normalized using Glyceraldehyde 3-phosphate dehydrogenase (GAPDH) and expressed in times versus control expression. Primer sequences can be provided under request.

**Western Blot Analysis**

Proteins were extracted from snap frozen liver tissues, resolved in sodium dodecyl sulfate–polyacrylamide gels and transferred to nitrocellulose membranes (Whatman). Whole cell lysates or nuclear proteins extracted after fractioning were run and blotted in nitrocellulose blotting membranes. Membranes were probed with FXR[4] or SIRT1[4, 6] primary antibody (Santa Cruz biotechnologies). As a loading control, we used Tubulin (Abcam) or Lamin B (Santa Cruz biotechnologies) antibodies for whole-cell or nuclear extracts respectively. As secondary antibodies, we used anti-mouse IgG–HRP-linked or anti-rabbit-IgG–HRP-linked (Santa Cruz biotechnologies).

**Immunoprecipitation**

FXR was immunoprecipitated using magnetic beads (Dynabeads®) following manufacturer’s instructions (Invitrogen). Whole liver protein lysates were extracted using RIPA buffer and incubated with magnetic beads previously bound and crosslinked with a FXR antibody (Santa Cruz biotechnologies). Proteins were immunoprecipitated by an o/n at 4C incubation with the crosslinked beads and were further eluted after incubation with 2x Laemmli buffer at 95C for 5min. Western blot analysis was performed as detailed above using Acetyl-lysine (Cell signaling) and FXR (Santa Cruz biotechnologies) antibodies.

**Flow Cytometry**

Immune cells were isolated from liver tissues after digestion with collagenase and successive washes and Percoll gradient. Isolated immune cells were stained with CD45-APC-Cy7 (BD), CD11b-PE (BD) and F4/80-FITC (Myltenyi) antibodies. Flow cytometry analysis was performed using BD LSRFortessa and analysed using FlowJo software.

**SUPPLEMENTAL FIGURE LEGENDS**

**Supplemental Figure 1. SIRT1 is up-regulated after BDL but not after Sham surgery** (A) Quantification of western blot analysis using Image Lab software (BioRad). (B) Western blot analysis of liver nuclear extracts from Sham operated WT animals at 3d and 7d after surgery showing no differences when compared to control untreated mice. (C) Quantification of western blot analysis using Image Lab software (BioRad). (D) Western blot analysis of WT and Mdr2^-/-^ livers followed by quantification. Values are mean ± SEM

**Supplemental Figure 2. SIRT1 expression is up-regulated in response to increased bile acid load and correlates with increased apoptotic cell death** (A) qPCR analysis of SIRT1 expression on isolated primary hepatocytes 3h after stimulation with different doses of bile acids (B) Quantification of caspase 3 activity in cell protein lysates evidenced increased apoptosis in response to bile acids. (C) qPCR analysis of SIRT1 expression on primary hepatocytes that were treated with a Caspase3 inhibitor (25 uM of Z-DEVD-FMK) 1h before addition of bile acids.(D) Western blot analysis and further quantification (ImageLab software) showing AMPK phosphorylation after CDCA and DCA treatment. (E) qPCR analysis of SIRT1 expression on primary hepatocytes that were treated with Compound C (50uM CC) 1h before addition of bile acids. (F) Western blot analysis and further quantification (ImageLab software) showing AMPK phosphorylation after CDCA and DCA treatment in hepatocyte cultured without (0%) or with (10%) FBS. (H) qPCR analysis of SIRT1 expression on primary hepatocytes cultured in the presence of 10% FBS treated with bile acids.

Values are mean ± SEM *In vitro* experiments were performed three times, each in duplicate. ***P* <0.01 [WT untreated vs WT treated with bile acids/CC).

**Supplemental Figure 3. SIRT1 upregulation correlates with increased apoptotic cell death, inflammation and fibrosis after BDL** (A)

(A) qPCR and (B) western blot analysis and quantification (ImageLab software) showing increased SIRT1 expression in livers from SIRT^oe^ mice compared to WTs (C) Quantification of caspase 3 activity in liver protein lysates and (D) TUNEL positive cells (% of positive cells vs % of DAPI positive nuclei ppf) evidenced increased apoptosis in SIRT^oe^ mice compared to WT after BDL. (E) Caspase 3 activity was determined in primary hepatocytes isolated from WT and SIRT^oe^ mice and cultured in the presence of CA, GCA. (F) qPCR analysis of TNFα and CCL expression. (G) Sirius Red and αSMA IHC on liver sections, morphometric quantification using Frida software and (H) qPCR analysis of Colagen1A1, αSMA and TGFβ in SIRT^oe^ and WT mice after BDL. All images are representative of original magnification 10x. Images are representative of n≥5 animals/time point; Values are mean ± SEM. *P <0.05, **P <0.01 [WT vs SIRT].

**Supplemental Figure 4. Sham surgery has no impact on serum liver damage markers, liver parenchyma, cell death, inflammation or fibrosis in WT and SIRT^oe^ mice.** (A) Blood transaminase profiles of WT and SIRT^oe^ animals, (B) H&E staining of liver sections from WT and SIRT^oe^ animals, (C) Caspase 3 activity determined on liver extracts showing that Sham surgery has no effect on liver parenchyma status or cell death (D) FACS analysis on liver isolated immune cells and (E) qPCR analysis showed no significant differences between control and sham operated WT and SIRT^oe^ mice. (F) Liver fibrogenesis was characterised by Sirius Red staining on liver sections (left panels) and αSMA IHC (right panels) from mice after BDL. (G) Quantification using Frida software of Sirius Red staining on liver sections from mice after sham surgery, expressed in % of positive staining per power field (ppf). (H) qPCR analysis of fibrosis markers (Images are representative of n≥5 animals/time point; Values are mean ± SEM. **P* <0.05, ***P* <0.01 [WT *vs* SIRT^oe^]) § p<0.05 [SIRT^oe^ *vs* Sham SIRT^oe^], ‡ *P* <0.05 [Sham/WT *vs* BDL/WT]) † *P* <0.05 [WT *vs* Sham/WT]) ## *P* <0.01 [Sham/SIRT^oe^ *vs* BDL/SIRT^oe^].

**Supplemental Figure 5. Sham surgery has no impact on FXR-signaling or bile acid transporters expression in WT and SIRT^oe^ mice.** (A) Faecal bile acids after BDL determined by MS-HPLC in WT and SIRT^oe^ mice after BDL (B) Western blot analysis of FXR expression, (C) qPCR analysis of SHP, CYp7A1, and (D) bile acid transporters in control, sham and BDL mice Values are mean ± SEM. **P* <0.05, ***P* <0.01, *** *P* <0.001 [WT *vs* SIRT^oe^]) † *P* <0.05 [WT *vs* Sham/WT]) ‡ *P* <0.05 [Sham/WT *vs* BDL/WT]).

**Supplemental Figure 6. Impact of 0.1%DDC diet in SIRT^oe^ mice.** (A) Blood transaminase profiles of WT and SIRT^oe^ animals, (B) H&E staining of liver sections from WT and SIRT^oe^ animals, (C) Caspase 3 activity determined on liver extracts and (D) TUNEL assay on liver sections showing that 0.1%DDC leads to aggravated liver injury in SIRT^oe^ animals (E) ductular reaction determined by CK19 immunostaining. (F) Liver fibrogenesis was characterised by Sirius Red staining on liver sections and morphometric analysis was done using Frida software and expressed in % of positive staining per power field (ppf). Images are representative of n≥5 animals/time point; Values are mean ± SEM. **P* <0.05, ***P* <0.01, *** *P* <0.001 [WT *vs* SIRT^oe^]).

**Supplemental Figure 7. *In vivo* and *in vitro* characterisation of hepatic cell death in hepatocyte-specific SIRT1 deleted mice** (A) qPCR and (B) western blot analysis and further quantification (ImageLab software) showing decreased SIRT1 expression in livers from SIRT^hep-/-^ mice compared to WTs. Apoptosis determination by TUNEL assay (% of positive cells vs % of DAPI positive nuclei ppf) and (D) quantification of caspase 3 activity in liver protein lysates evidenced decrease apoptosis in SIRT^hep-/-^ mice compared to WT after BDL. (E) cell viability, via MTT assay, was determined in primary hepatocytes isolated from WT and SIRT^hep-/-^ mice and cultured in the presence of CDCA and DCA, indicating an increase in necrosis. (F) western blot analysis of phosphorylated AMPK in primary hepatocytes treated with CDCA and DCA followed by quantification using ImageLab software. (G) Hepatocytes treated with Compound C (50uM) 1 hour before stimulation with bile acids. Values are mean ± SEM *In vitro* experiments were performed three times, each in duplicate.

**Supplemental Figure 8. *Hepatocyte-specific SIRT1 depleted mice show similar ductular reaction, inflammation and fibrosis after BDL compared to WT littermates.*** (A) qPCR analysis of TNFα and CCL2 expression, (B) CK19 immunostaining and (C) further morphometric quantification using Frida software, (D) Sirius Red staining and (E) αSMA IHC, respective quantification and (F) qPCR analysis of fibrosis markers supported that BDL had a comparable impact on WT and SIRT^hep-/-^ mice. Images are representative of n≥5 animals/time point; Values are mean ± SEM of n≥5 animals/time point.

**Supplemental Figure 9. FXR is deacetylated after BDL.** (A) Western blot analysis and (B) further quantification (ImageLab software) of immunoprecipitates of liver extracts using a FXR antibody showing decrease acetylation after BDL in WT mice and SIRT^hep-/-^ animals (Images are representative of n≥3 animals/time point).

**Supplemental Figure 10. Sham surgery has no impact on serum liver damage markers, liver parenchyma, cell death, inflammation or fibrosis in WT and SIRT^hep-/-^ mice.** (A) Blood transaminase profiles of WT and SIRT^hep-/-^ animals, (B) H&E staining of liver sections from WT and SIRT^hep-/-^ animals, (C) TUNEL assay on liver sections and (D) Caspase 3 activity determined on liver extracts showing that Sham surgery has no effect on liver parenchyma status or cell death (E) FACS analysis on liver isolated immune cells and (F) qPCR analysis showed no significant differences between control and sham operated WT and SIRT^hep-/-^ mice. (G) CK19 IHC on liver sections (H) Liver fibrogenesis was characterised by Sirius Red staining on liver sections (left panels) and aSMA IHC (right panels) from mice after BDL, quantification using Frida software of Sirius Red staining on liver sections from mice after sham surgery, expressed in % of positive staining per power field (ppf). (I) qPCR analysis of fibrosis markers (Images are representative of n≥5 animals/time point; Values are mean ± SEM. **P* <0.05 [WT *vs* SIRT^oe^]).

**Supplemental Figure 11. Impact of 0.1%DDC diet in SIRT^hep-/-^ mice.** (A) Blood transaminase profiles of WT and SIRT^hep-/-^ animals, (B) H&E staining of liver sections from WT and SIRT^hep-/-^ animals, (C) Caspase 3 activity determined on liver extracts and (D) TUNEL assay on liver sections showing that 0.1%DDC leads to comparable degree of liver injury in SIRT^hep-/-^ animals compared to WT littermates (E) ductular reaction determined by CK19 immunostaining was found to be moderately increased in DDC/SIRT^hep-/-^ but was not significantly different from WT mice. (F) Liver fibrogenesis was characterised by Sirius Red staining on liver sections and morphometric analysis was done using Frida software and expressed in % of positive staining per power field (ppf). (G) Western blot analysis showing FXR expression at 1 week after DDC diet. Images are representative of n≥5 animals/time point; Values are mean ± SEM. **P* <0.05 ***P* <0.01 [WT *vs* SIRT^hep-/-^]).

**Supplemental Figure 12. NorUDCA reduces SIRT expression, attenuates apoptosis and fibrosis after BDL in SIRT^oe^ mice** (A) qPCR on liver tissue samples obtained from SIRT and NorUDCA/SIRT mice before and 1day and 14days after BDL. (B) quantification of SIRT1 and (C) FXR western blot analysis using ImageLab software (BioRad). (D) Quantification of bile acid pool size in livers by HPLC showing reduction of bile acid pool size in NorUDCA/SIRT^oe^ mice after BDL. (E) Quantification of TUNEL positive hepatocytes compared to total nuclei ppf represented in %. (F) qPCR analysis of fibrosis related genes. (G) Immunohistochemistry using an anti-SIRT1 Ab in liver sections from 8-week old Mdr2^-/-^ and NorUDCA-fed Mdr2^-/-^ mice and quantification of the % of positive hepatocytes for SIRT1. (H) Western blot analysis of nuclear extracts showing reduced SIRT1 expression in norUDCA/Mdr2^-/-^ mice compared to Mdr2^-/-^ animals. Values are mean ± SD. n≥5 animals/time point; **P* <0.05, ***P* <0.01 [SIRT *vs* NorUDCA/SIRT]). n=3-5 fields counted per n=5 Mdr2^-/-^ mice and n=6 NorUDCA/Mdr2^-/-^. ***P* <0.01 [WT control *vs* Mdr2^-/-^].

**REFERENCES**

[1] Herranz D, Munoz-Martin M, Canamero M, Mulero F, Martinez-Pastor B, Fernandez-Capetillo O, et al. Sirt1 improves healthy ageing and protects from metabolic syndrome-associated cancer. Nat Commun 2010;1:3.

[2] Fernandez-Alvarez S, Gutierrez-de Juan V, Zubiete-Franco I, Barbier-Torres L, Lahoz A, Pares A, et al. TRAIL-producing NK cells contribute to liver injury and related fibrogenesis in the context of GNMT deficiency. Lab Invest 2015;95:223-236.

[3] Beraza N, Ofner-Ziegenfuss L, Ehedego H, Boekschoten M, Bischoff SC, Mueller M, et al. Nor-ursodeoxycholic acid reverses hepatocyte-specific nemo-dependent steatohepatitis. Gut 2011;60:387-396.

[4] Garcia-Rodriguez JL, Barbier-Torres L, Fernandez-Alvarez S, Gutierrez-de Juan V, Monte MJ, Halilbasic E, et al. SIRT1 controls liver regeneration by regulating bile acid metabolism through farnesoid X receptor and mammalian target of rapamycin signaling. Hepatology 2014;59:1972-1983.

[5] Halilbasic E, Fiorotto R, Fickert P, Marschall HU, Moustafa T, Spirli C, et al. Side chain structure determines unique physiologic and therapeutic properties of norursodeoxycholic acid in Mdr2-/- mice. Hepatology 2009;49:1972-1981.

[6] Dong S, Jia C, Zhang S, Fan G, Li Y, Shan P, et al. The REGgamma proteasome regulates hepatic lipid metabolism through inhibition of autophagy. Cell Metab 2013;18:380-391.
